# Experimental Investigation on the Detection of Multiple Surface Cracks Using Vibrothermography with a Low-Power Piezoceramic Actuator

**DOI:** 10.3390/s17122705

**Published:** 2017-11-23

**Authors:** Changhang Xu, Jing Xie, Wuyang Zhang, Qingzhao Kong, Guoming Chen, Gangbing Song

**Affiliations:** 1 College of Mechanical and Electronic Engineering, China University of Petroleum, Qingdao 266580, China; xiejing@upc.edu.cn (J.X.); 15666139907@163.com (W.Z.); gmchen@upc.edu.cn (G.C.); 2 Department of Mechanical Engineering, University of Houston, Houston, TX 77004, USA; gsong@uh.edu

**Keywords:** vibrothermography, multiple cracks detection, lower power actuator, piezoceramic actuator

## Abstract

Vibrothermography often employs a high-power actuator to generate heat on a specimen to reveal damage, however, the high-power actuator brings inconvenience to the application and possibly introduces additional damage to the inspected objects. This study uses a low-power piezoceramic transducer as the actuator of vibrothermography and explores its ability to detect multiple surface cracks in a metal part. Experiments were conducted on a thin aluminum beam with three cracks in different orientations. Detailed analyses of both thermograms and temperature data are presented to validate the proposed vibrothermography method. To further investigate the performance of the proposed vibrothermography method, we experimentally studied the effects of several critical factors, including the amplitude of excitation signal, specimen constraints, relative position between the transducer and cracks (the transducer is mounted on the same or the opposite side with the cracks). The results demonstrate that all cracks can be detected conveniently and simultaneously by using the proposed low-power vibrothermography. We also found that the magnitude of excitation signal and the specimen constraints have a great influence on detection results. Combined with effective data processing methods, such as Fourier transformation employed in this study, the proposed method provides a promising potential to detect multiple cracks on a metal surface in a safe and effective manner.

## 1. Introduction

Active infrared thermography (IRT) has received increasing attention in recent years due to its advantages of non-contact detection, high efficiency, and clear visualization. A few kinds of heating excitations are commonly used in active IRT to detect defects [1,2,3,4,5]. Based on different heating excitations, IRT techniques can be classified as pulsed phase thermography [6], step heating thermography [7], lock-in thermography [8], microwave thermography [9], laser thermography [10], inductive thermography [11], and vibrothermography. Among these techniques, vibrothermography, also known as ultrasonic thermography or thermosonics, is a nondestructive testing (NDT) technique to detect the defects in materials using the combination of mechanical vibration and infrared imaging. The basic concept of vibrothermography relies on the conversion of mechanical energy to thermal energy [12]. When mechanical vibrations are introduced into a specimen, friction-induced, viscoelasticity-induced, or plastic deformation-induced heat will be generated around the defects, which can be recorded by an infrared camera to detect the defects. One major advantage of vibrothermography lies in the fact that only the defects contribute to the localized temperature rising, which makes it a faster and safer NDT technique [13]. Vibrothermography has been verified as a promising and effective method for detecting cracks in metals, as well as impact, delamination, or debonding in composite materials [14,15,16,17,18,19,20].

In recent years, the surface crack detection in metal parts using vibrothermography has become one of major research interests. The related works have been focused on the investigations of detection mechanisms, analysis of influential factors and defect characterization methods. In the current investigations, high power excitation devices are usually used to generate heat in the specimen with a single crack. To make the testing work more convenience and to avoid introducing new damage to the tested specimen, it is necessary to investigate vibrothermography with a low-power actuator to detect multiple cracks on metal parts. Recently, there has been increasing interest in using vibrothermography with low-power actuator to detect defects in composite materials, however, the related investigations on metal components are quite limited. In the previous work, we developed a coupled thermo-electro-mechanical analysis with implicit finite element method to simulate a low power piezoceramic ultrasonic actuator and the corresponding heat generation in metallic plates with multiple surface cracks [21], which only involved a simple experiment to demonstrate the presented finite element model. Aiming at further investigating the feasibility of using vibrothermography to detect multiple cracks on metal surface based on lower power actuator, this work implemented a detailed experimental investigation on the specimen used in [21]. The characterization of the cracks is analyzed through both the thermograms and the temperature response. Influences of several critical factors on the detection results are analyzed based on experimental results. Additionally, discrete Fourier transformation (DFT) is used as an image processing method to enhance the cracks features in the thermograms, which would improve the feasibility of the viborthermography with lower-power actuator for detecting surface cracks on metal parts. 

## 2. Brief Literature Review

Experimental and numerical researches have been carried out to develop an improved understanding for the detection mechanisms of vibrothermography. Holand et al. [22] revealed the fundamental physics governing the heat generation and verified the primary sources of heat generation in vibrothermography, namely the frictional rubbing, the plastic deformations and the viscoelasticity. Mabrouki et al. [23] presented a finite element model to investigate the mechanism of crack detection using vibrothermography based on the plastic deformation heating concept. Numerical results indicated that the conversion of plastic work to heat occurs when the von Mises stress on the crack faces exceeds the yield stress of the material under vibration. Additionally, their experimental results showed that the generated heat can be detected around cracks about 1 mm. Rizi et al. [24] also developed two finite element models for simulating the process of vibrothermography to qualitatively study the crack heating caused by internal and external energy losses, respectively. The local friction between crack surfaces was considered to construct the model of external loss while the viscoelastic and the thermo-elastic damping were employed for modelling the internal loss. In [25], it was revealed that the vibration-induced heat generation might be accompanied with tribological damage which partially resulted in the non-repeatability of vibrothermographic testing. To explore the damping-related viscoelastic heat generation mechanism of vibrothermography, Montanini et al. [26] studied the relationship among the vibrational mode shapes, ultrasonic excitation frequency, and other factors. Feng et al. [27] presented a mathematical heat transfer model to compute the temperature distribution of the metallic surface with a crack and found that there exists a quantitative relation between the heat source depth and the ratio of the temperature response of the crack vicinity at the opposite side to that of the front side.

In addition to the detection mechanisms, the influential factor is another major research issue of vibrothermography. Holland et al. [28] stated that the heat generation increases with the increasing of frequency during detecting of fatigue cracks. Zhang et al. [29] described the relationship between the engagement force and the vibration characteristic of vibrothermography based on a hyperbolic curve model. Bolu et al. [30] compared several measuring methods of excited vibration to calculate a heat index, which represents the amount of vibration energy. 

Recently, qualitative and quantitative methods to characterize the cracks detected by using vibrothermography have been developed. Holland et al. [31] performed a quantitative evaluation on the relationship between the temperature distribution and the crack dimensions. Based on the matched filter technique, they also conducted investigations on the characterization of cracks with automatic crack detection [32] and a thermographic signal reconstruction [33]. Mendioroz et al. [34] proposed a stabilized inversion algorithm combined with the experimental data to characterize the vertical cracks. A defect identification method based on the parameter estimation of a gray-level histogram to automatically extract the quantitative features of the cracks from thermogram sequences in [35]. Feng et al. [36] investigated the relationship between temperature rising at a fatigue crack and the test conditions. The results showed that engagement force and excitation intensity have significant influences on the temperature rising, and the optimized test conditions can be determined based on the probability of detection (POD) and probability of alarm (POA). Additionally, some investigations were performed on the structural vibration behavior of specimens for vibrothermography, which show there exists relationships between the vibration response (strain, displacements) and the temperature response due to the hysteretic heating generated by viscoelastic or frictional heating [37].

The power of excitation is one of the key factors for defect detection using vibrothermography. In the above-mentioned applications, high power (up to 2 kW) excitation devices, such as ultrasonic welding horns, are the usual choices of excitation sources. However, high power excitation is not necessary to guarantee enough heat generated around the cracks due to the nonlinear coupling between the horn and specimen [38]. Moreover, it should be noted that the chaotic vibration of the specimen can be easily caused by ultrasonic welding horn, which might make the crack detection more difficult and introduce new potential risks of damage to the specimens [39]. Therefore, there is a need for investigation of vibrothermography based on low-power actuators, which can generate less but enough heat for detecting surface cracks on metal parts. There are a few reported works on vibrothermography with low-power actuator, but most of them focus on composite sample rather than metal parts. Balageas et al. [40] utilized an adhesive bonded piezoelectric disc to perform lock-in detection on a carbon fiber reinforced polymer (CFRP) plate. The results showed the effectiveness of the detection based on low-power piezoelectric disks depends on the optimization of the exciter parameters, such as the excitation frequency, the modulation frequency, and the amplitude. Kang et al. [41] pointed out that a low-power PZT transducer coupled with wax can be used as a vibration exciter of vibrothermography for detecting the damage on a CFRP composite plate. Rahammer [42] used a combination of repeated sweeping excitation signal and Fourier-transforming the temperature data to enhance the very noisy temperature data, which was demonstrated effective to detect the defects on the CFRP specimens with low power excitation (50 V). In general, current research mainly focuses on detecting delamination or impact damage in composite parts, which is different from the cracks detection for metal parts in terms of materials properties and defect types. Consequently, the feasibility of vibrothermography with low-power actuator to detect cracks on metal parts needs further investigations.

Moreover, vibrothermography has been only verified efficient to detect single surface crack in metal parts. For multiple surface cracks in metal parts, to the authors’ knowledge, limited research of vibrothermography has been found. Different from detecting single cracks, detection of multiple surface cracks using vibrothermography is more complicated due to the interference among different cracks with same or different orientations. Since this interference might play significant roles on the mechanical wave propagation, which probably affects the detectability of vibrothermography. Hence, it is still necessary to investigate the detectability of vibrothermography with a low-power actuator for multiple surface cracks detection on metal parts.

## 3. Experimental Setup

**Figure 1:**
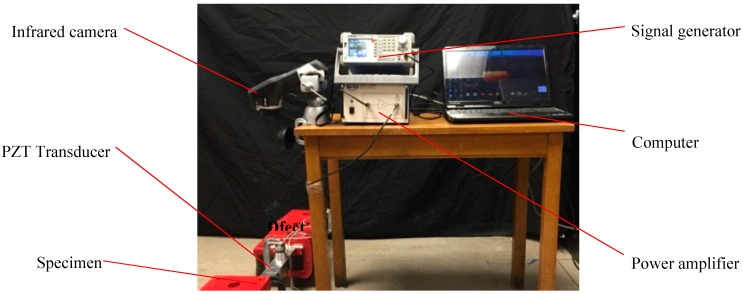
The experimental setup for multi cracks detection using vibrothermography.

**Figure 2:**
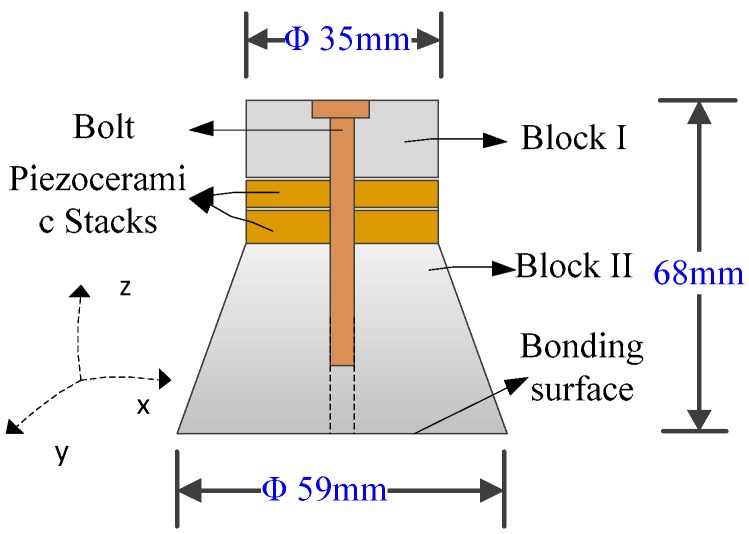
A PZT transducer as an ultrasonic exciter.

The experimental setup is shown in Figure 1, which includes the following major parts: a test specimen, a signal generator, an ultrasonic exciter, an amplifier for the ultrasonic exciter, an infrared camera, and a computer that hosts the infrared camera. In this system, we used a simple PZT transducer as the ultrasonic exciter, as shown in Figure 2. The PZT transducer is assembled as a sandwiched structure including two 2 mm thick piezoceramic disks, which are clamped with two steel blocks by a bolt. Unlike ultrasonic welding horn commonly used by many literatures, the actuator does not concentrate the ultrasonic power on a tipped area. The ultrasonic power generated by PZT are distributed on a circular surface, which introduces the ultrasonic power to the specimen in a unique and safe manner, as shown in Figure 2. The contact circular surface has a diameter of 59 mm. Combining the signal generator and power amplifier, different types of signals with a maximum frequency of 2 MHz and a maximum amplitude of 150 V can be generated for the actuator.

**Figure 3:**
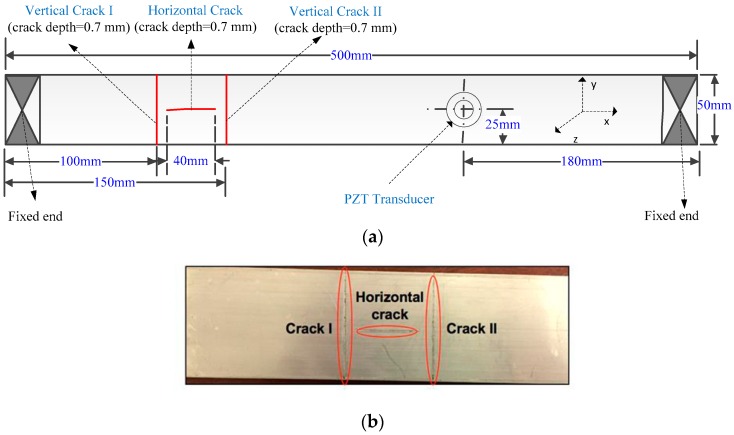
Test specimen with thickness of 3 mm: (**a**) the sketch of the specimen; and (**b**) a zoomed view of the cracks.

A thin aluminum beam with a dimension of 500 mm × 100 mm × 3 mm was used as the test specimen in this work. There are three cracks with different orientations on the surface of the specimen, as shown in Figure 3. During the experiments, the specimen was fixed at one or both ends with clamps. The PZT transducer was mounted on the same side and the opposite side with the cracks, respectively. Then, the transducer, which was controlled by the signal generator with a power amplifier, was used to excite the specimen at different locations to vibrate along the z-axis with different frequencies and amplitudes. An IR camera (FLIR E60 of FLIR Systems, Inc., Wilsonville, OR, USA) was consistently placed on the same side with the crack to record the temperature variation of the specimen surface. It has a resolution of 320 × 240 pixels at a frame rate of 30 Hz and a sensitivity of 0.01 °C.

## 4. Results and Discussions

In this section, we present the experimental results of multiple surface cracks detection using vibrothermography with the low-power actuator described in Section 3. Feature analyses of cracks are performed on both thermograms and temperature data to demonstrate the effectiveness of the detection technique. In addition, the influences of several factors are also discussed, including the amplitude of the excitation signal, specimen constraints, and the relative position between the transducer and cracks.

### 4.1. Thermogram Analysis

**Figure 4:**
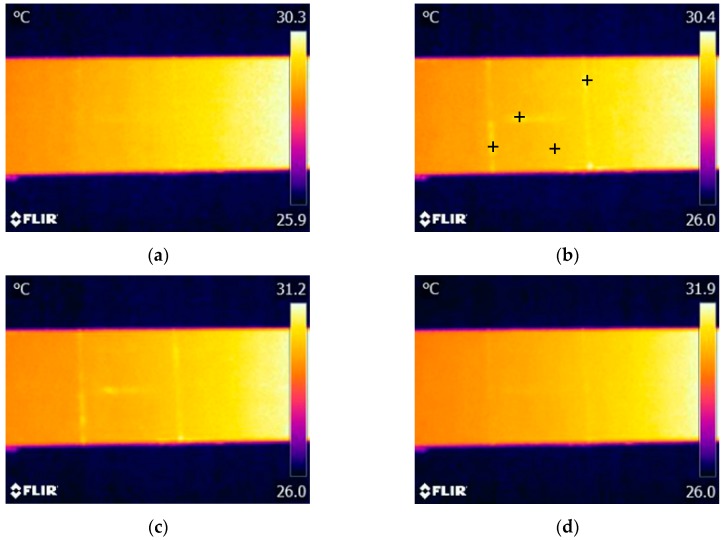
With the excitation amplitude of 150 V, the thermograms captured at: (**a**) 4.8 s; (**b**) 6 s; (**c**) 45 s; and (**d**) 92 s. Note: The actuator was mounted on the right part of the specimen, which is not displayed in these figures.

Measurements of electrical impedance of the piezoelectric transducer were performed prior to the vibrothermography test, as mentioned in [21]. It was found that the minimum impedance frequency (resonance frequency) of the piezoelectric transducer used in our experiments is around 40.7 kHz. This frequency was then used as the excitation frequency because under a sinusoidal wave with such frequency the transducer has a maximum output. The infrared camera was operated at its maximum frame frequency of 30 Hz. A total of 2850 thermogram frames were sequentially recorded for each test (recording time of 95 s). Each thermogram sequence was divided into three periods: frames from 1 to 150 (0–5 s) as the stage before excitation; frames from 151 to 2700 (5–90 s) as the excitation stage; and frames from 2701 to 2850 (90–95 s) as the stage after excitation. Figure 4 shows four typical thermograms recorded at each stage when the excitation signal amplitude was set as 150 V, which is the maximum amplitude generated by the experimental system.

As shown in Figure 4a, it is difficult to identify the cracks from the thermogram before vibration excitation due to the poor thermal contrast between the cracks and the surrounding areas. However, all three cracks immediately showed distinguished features in the thermogram once the vibration was generated, as shown in Figure 4b. Each crack is indicated as a bright region in this thermogram, which demonstrates a temperature rising occurs at the crack region. By comparing Figure 4b,c, we can find that the temperature risings of cracks have a slight increasing trend during the exciting stage. All crack indications immediately disappear once the excitation is stopped (Figure 4d). Consequently, these experimental results demonstrate that the used ultrasonic transducer is effective for simultaneously generating enough heat at the locations of the three cracks, which makes it feasible to detect multiple cracks on the aluminum beam by using vibrothermography with a low-power actuator. It should also be noticed that the temperature of the right side of the specimen is always higher than that of the left side. The reason lies in that after conducting several tests, the body temperature of the actuator is quite high, which causes heat conduction from the mounting position of the actuator (right side) to the location of the cracks (left side) during the experiments.

It is important to understand the thermal pattern of each crack. From Figure 4b,c, it can be observed that once the excitation starts, each crack presents as a brighter region while the other regions are unchanged, which then results in a large thermal contrast between each crack and its surrounding regions. Therefore, the heat is only generated in the crack regions rather than on the entire surface of the specimen, which makes the crack boundaries quite distinct in the thermograms. These clear crack boundaries are important to quantitatively characterize a crack, such as describing the location, length, and shape of a crack. In addition, we can find that there are some subtle differences between the indications of the vertical cracks and that of the horizontal one. The vertical cracks are indicated with more distinct features compared to the horizontal one. This may be caused by the relationship between the direction of mechanical vibration wave propagation and the crack orientation.

### 4.2. Temperature Data Analysis

**Figure 5:**
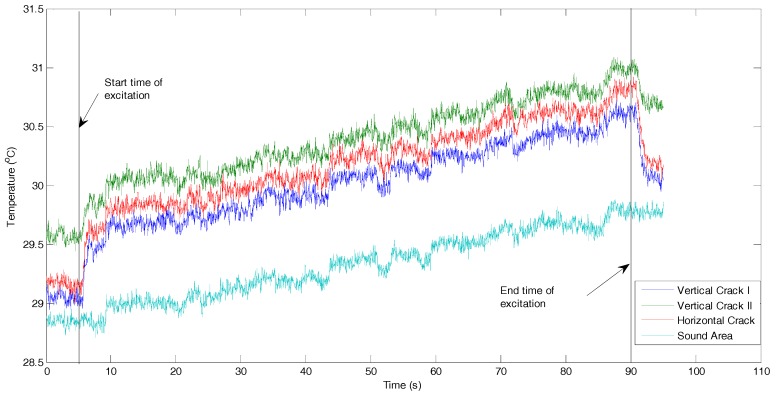
Temperature evolutions of the cracks and surrounding areas (the locations of the points are shown in Figure 4b).

Here we discuss the measured temperature data to reveal more information for characterization of the cracks. Figure 5 illustrates the temperature responses of four spots on the three cracks and the surrounding area, respectively. The locations of four spots are shown in Figure 4b. It can be found that the temperature responses of three cracks changed with the same trend during the entire experimental process: sharply temperature increasing (about 0.3–0.5 °C within 1–2 s) at the beginning of the excitation, steadily increasing during the excitation stage, and then the temperature sharply decreases (about 0.3–0.5 °C within 1–2 s) at the end of the excitation. However, such a sharp increase and sharp decrease cannot be found in the temperature response of the surrounding area. The temperature response of the surrounding area steadily and monotonically increase during the entire period, which is caused by the heat conduction from the transducer mounted on the right side. Consequently, these temperature responds indicate that by using the low-power actuator, significant temperature increases will promptly occur at the crack locations.

As shown in Figure 5, the pattern of temperature changes for a horizontal crack is extremely similar to those of the two vertical cracks. It can be found that the vibration introduced by the PZT transducer can simultaneously generate enough heat for all surface cracks in different orientations. It indicates that the detection result of vibrothermography is not sensitive to the orientation of the crack on the specimen used in this study. The same results were also obtained in our previous numerical study [21]. Therefore, vibrothermography with the low-power actuator can detect multiple surface cracks on a thin aluminum beam in a simultaneous and effective manner.

Therefore, the proposed low-power actuator based heating excitation can simultaneously generate obvious temperature risings in all cracks at different locations and in different orientations, which contributes to detect multiple surface cracks on such thin aluminum beam-like metal parts by using vibrothermography in a fast, effective, and simultaneous manner.

### 4.3. Analysis of Influencing Factors

In order to further investigate the effectiveness of vibrothermography with low-power actuator to detect multiple cracks on metal parts, here we analyze the influences of three significant factors on the detection results. These factors include the excitation signal amplitude, the constraint condition of the test object, and the relative position between the excitation and cracks (same or opposite side).

**Figure 6:**
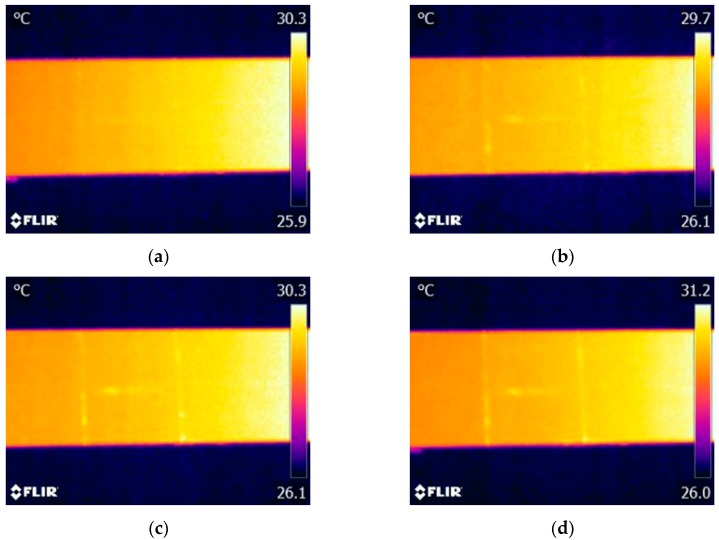
IR image of different excitation signal amplitudes: (**a**) 60 V; (**b**) 80 V; (**c**) 120 V; and (**d**) 150 V.

A set of experiments were conducted to study the effect of the excitation signal amplitude on the cracks features detected in thermograms. Figure 6 shows the thermograms corresponding to excitation amplitudes of 60 V, 80 V, 120 V, and 150 V, respectively. We observe that all cracks can be visually detected from the raw thermograms in case that the excitation amplitude is not less than 80 V. When the excitation amplitude is within the range of 80 V–150 V, the corresponding power of the PZT transducer is approximately within a range of 5 W–14 W. This power range is much lower compared to the power of an ultrasonic welding horn used in many literatures (up to 2 kW). To evaluate the defect visibility in each thermogram, here we discuss the thermal contrasts of cracks, which is defined as the temperature difference between the crack and its surrounding areas. Since all three cracks cannot be visually detected in the case of an excitation amplitude of 60 V, as shown in Figure 6a, only the thermal contrasts of cracks in thermograms of 80 V, 120 V, and 150 V are listed in Table 1. We can find from Table 1 that the thermal contrast of each crack proportionally increases with the increasing of excitation amplitude. Therefore, with the increase of the excitation amplitude, the crack can be indicated more clearly.

**Figure 7:**
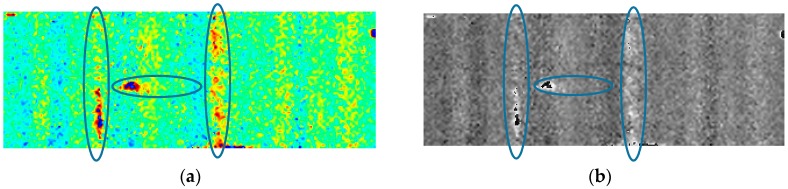
Phase image at 0.043 Hz obtained by implementing DFT on the raw detection data with using an excitation amplitude of 60 V: (**a**) color mode; and (**b**) grayscale mode.

As stated previously, the cracks cannot be visually detected from Figure 6a corresponding to the excitation amplitudes of 60 V due to its poor thermal contrast. Here we aim to use image processing methods to acquire more distinct crack indications, which is meaningful for damage detection with vibrothermography based on lower power actuator. Discrete Fourier transformation (DFT) has been widely used in pulsed thermography, which constructs the technique named as pulsed phase thermography (PPT). In this technique, thermal variation signal of each pixel is sequentially processed by DFT. Then, the acquired phase values and amplitude values on each frequency are employed to construct a phase image and an amplitude image for each frequency. Detailed fundamental of DFT can be found in [43]. We implemented DFT on the raw detection data acquired with 60 V as the excitation amplitude, the corresponding current was about 50 mA. From Figure 6a, we know that, with this excitation power, cracks cannot be identified through the raw thermogram. The contrast between cracks and the surrounding sound regions are too weak to be observed. After implementing DFT, the acquired phase image corresponding to the frequency of 0.043 Hz is shown in Figure 7a with a color mode and in Figure 7b in grayscale mode, respectively. By comparing these two images to the raw thermogram shown in Figure 6a, we can find that, after implementing DFT, the vertical crack II can be definitely identified through the phase image. The indication of the vertical crack I is also significantly improved in the phase images, although the upper part of it is still invisible. Additionally, compared to the raw thermogram, the phase image also presents more distinct features for the left end of the horizontal crack. This indicates that feature enhancement can be achieved to different degree for all cracks in the reconstructed image shown as Figure 7. In our experiment, the maximum instant power of the piezoceramic actuator was only about 3 W (60 V and 50 mA), which is significantly lower than the commonly used power in the literature. Consequently, by employing appropriate data processing methods, successful detection with lower excitation power can be expected for our actuator.

**Figure 8:**
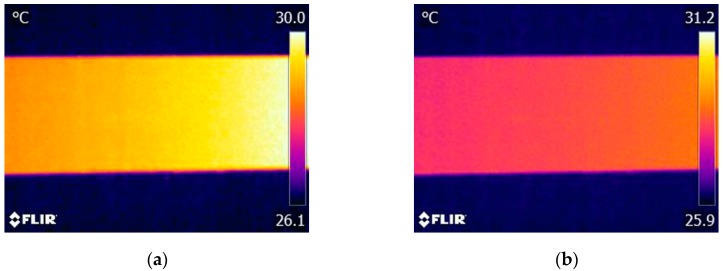
Thermograms of specimen captured under different constraints conditions: (**a**) partially fixed at both ends; and (**b**) fixed at one end.

According to the fundamental of vibrothermography, heat energy generated around the cracks is closely related to the propagation of the mechanical wave. The constraint condition of the test object is a critical factor which affects the behavior of the mechanical wave propagation. Hence, it is necessary to investigate the possible effects of specimen’s constraint condition on crack detection. To the specimen used in this work, we considered three constraint conditions: rigidly fixed at both ends, partially fixed at both ends (the specimen was not very firmly fixed), and fixed at only one end (the other end is free). Figure 8 shows the captured thermograms under the latter two constraint conditions with the excitation amplitude of 150 V. The experimental results corresponding to the first condition with the same excitation power have been shown in Figure 6d. Comparing these experimental results for the three constraint conditions (Figure 6d and Figure 8), we can find that only by rigidly fixing the specimen at both ends, all cracks can be successfully detected with this excitation power. Other two constraint conditions cannot provide efficient detection results. The possible main reason lies in that, for the thin aluminum beam studied in this research, the fixed constraint condition can reflect more mechanical wave introduced by PZT transducer than the free-constraint condition, which leads to more mechanical energy converting to thermal energy. On the contrary, such a vibration will preferentially convert to free vibration of the specimen rather than thermal energy at the cracks in the case of the free-constraint conditions. This experimental result suggests that, when applying vibrothermography to detect surface cracks on a thin metal beam, keeping the specimen under an appropriate constraint condition is essential for an effective detection.

**Figure 9:**
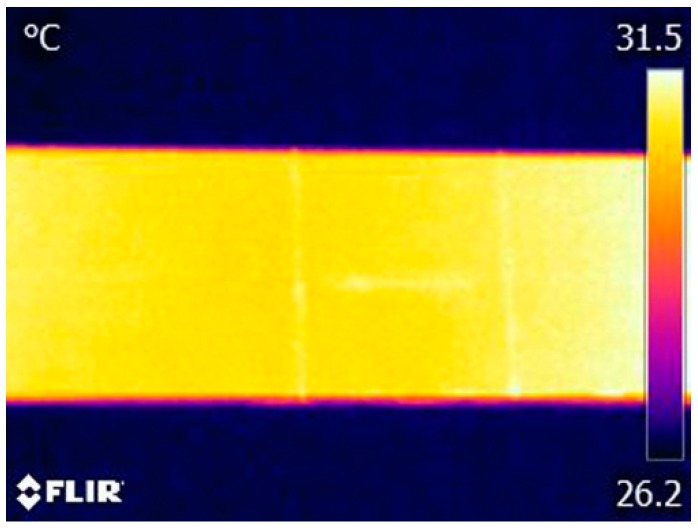
IR image with the actuator mounted on the opposite side of the cracks and IR camera.

Finally, to further demonstrate the feasibility of the developed low-power vibrothermography, we experimentally investigated the influence of the PZT transducer position on the detection result. The results shown above (Figure 4, Figure 5, Figure 6, Figure 7 and Figure 8) come from experiments in which the PZT transducer was mounted on the same side as the cracks. Hence, here we conducted additional experiments by mounting the transducer at the back surface, which is opposite to the surface with cracks. The IR camera was still located the same side with the cracks during the experiments. All the other parameters were set the same as those in the above experiments, including the excitation signal magnitude (80 V), the distance between actuator and cracks (Figure 3), and the constraint condition of the specimen (rigidly fixed at both ends). Figure 9 shows the thermogram captured at beginning of excitation stage. We can find that under this detection condition, all three cracks can also be effectively detected. Compared with the thermogram recorded in case that the transducer was mounted at the crack surface (Figure 6b), Figure 9 shows the even more distinct crack boundaries. It indicates that the mechanical vibrations introduced into the thin beam specimen at one side also can induce enough heat energy around the cracks localized at the other side for defects detection. Hence, it is evident that, for such thin beam metal part, all the surface cracks studied in this work are detectable regardless on which side the PZT transducer is mounted on the specimen. Obviously, such a robustness of vibrothermography to the position of PZT transducer is important to ensure its feasibility for practical applications.

## 5. Conclusions

In this work, the effectiveness of using vibrothermography with a low-power ultrasonic actuator to detect the multiple surface cracks on a metallic beam is experimentally studied. This low-power actuator involves two piezoceramic disks that are clamped with two metal blocks tightened by a bolt. An experimental apparatus was set up and series of experiments were conducted on a thin aluminum beam with three cracks in different orientations. The experimental results show that by applying an excitation signal with an amplitude of 80 V, all three cracks in the thin aluminum beam with different orientations can be simultaneously detected from the raw thermograms within the excitation time of 1–2 s. We also analyze the influences of several factors on crack detection using vibrothermography. The results indicate that the detectability of cracks is closely related to the amplitude of excitation signal. When the amplitude is set as no less than 80 V, effective detection for all cracks can be obtained. Moreover, it is demonstrated that using image processing methods to enhance the features of cracks in raw thermograms is a promising way to achieve effective detection using vibrothermography with lower power (the excitation signal amplitude of 60 V in this work). It is also clearly revealed that vibrothermography is robust with respect to the factor of mounting the PZT transducer at the same location, but on the opposite side of the cracks. Finally, the experimental results suggest that the vibrothermography is sensitive to the constraint conditions of the specimen and the configuration of double fixed ends is found to be the best way for crack detection in the thin beam-like metal part presented in this research. However, this work presents only a preliminary attempt to investigate the effectiveness of vibrothermography with a low-power actuator to detect multiple cracks on metal parts. Combining with the numerical model presented in [21] and other effective image processing methods in thermography [44,45], future work will focus on further improving the feasibility of this technique for detecting surface cracks with varied dimensions or subsurface cracks on metal parts.

## Figures and Tables

**Table 1:** Temperature difference between each crack and its surrounding areas.

Crack	Cracks I			Cracks II			Horizontal Crack		
Voltage (V)	80	120	150	80	120	150	80	120	150
Temperature Difference (°C)	0.3	0.4	0.7	0.2	0.4	0.5	0.4	0.5	0.6
